# Seven-month-old infants detect symmetrical structures in multi-featured abstract visual patterns

**DOI:** 10.1371/journal.pone.0266938

**Published:** 2022-05-11

**Authors:** Irene de la Cruz-Pavía, Gesche Westphal-Fitch, W. Tecumseh Fitch, Judit Gervain

**Affiliations:** 1 Department of Linguistics and Basque Studies, Universidad del País Vasco/Euskal Herriko Unibertsitatea (UPV/EHU), Vitoria-Gasteiz, Spain; 2 Basque Foundation for Science Ikerbasque, Bilbao, Spain; 3 Integrative Neuroscience and Cognition Center, CNRS, Université Paris Cité, Paris, France; 4 Universität Wien, Vienna, Austria; 5 Department of Developmental and Social Psychology, Università di Padova, Padova, Italy; Max-Planck-Institut fur Kognitions- und Neurowissenschaften, GERMANY

## Abstract

The present study investigated 7-month-old infants’ ability to perceive structural symmetry in mosaic-like abstract visual patterns. We examined infants’ (n = 98) spontaneous looking behaviour to mosaic-like sequences with symmetrical and asymmetrical structures. Sequences were composed of square tiles from two categories that differed in their colour scheme and internal shape. We manipulated sequence length (3 or 5 tiles) and abstractness of the symmetry (token vs. category level). The 7-month-olds discriminated structurally symmetrical from asymmetrical mosaics in the first half of the test phase (first 8 trials). Sequence length, level of symmetry, or number of unique tiles per sequence did not significantly modulate infants’ looking behaviour. These results suggest that very young infants detect differences in structural symmetry in multi-featured visual patterns.

## Introduction

The oldest human marking found to date is an abstract zigzag pattern engraved on a shell, created by an early hominin, *Homo erectus*, half a million years ago in Java, Indonesia [1]. The earliest known drawing from our own species, *Homo sapiens*, is also abstract: a crisscross pattern engraved on ochre around 73,000 years ago from Blombos cave, South Africa [2]. This abstract drawing predates by about 30,000 years the earliest known figurative painting, a hunting scene discovered in a cave in Sulawesi, Indonesia [3]. These findings show that, from our earliest beginnings, humans have produced patterned abstract designs. Such designs can be found across cultures, ages, and media: in the Girih patterns used in Islamic art and architecture, in the textiles woven by the Incas, in the decoration of Celtic jewellery, in Chaco Canyon’s ceramics, in Maasai shields, or in modern quilt, wallpaper, or fabric designs.

Abstract visual patterns typically consist of basic units that are repeated and/or combined, and their arrangement in the plane can often be described by a set of rules or, in other words, a visual “grammar” [4, 5]. As a result of the structured combination of their elements, these visual designs are often symmetrical.

Symmetry of various sorts is ubiquitous in the world, characterizing objects, figures, and patterns that occur in nature (e.g. in physics, biology, chemistry) as well as in all fields of human creation (e.g. in music, art, poetry) [4]. Adults appear attuned to perceive symmetry in visual stimuli [6–8]: we detect and discriminate symmetry rapidly [9, 10], and remember symmetrical displays better than asymmetrical ones [11, 12]. Symmetry of background elements in a visual search task facilitates participants’ identification of a target, showing that symmetry is processed automatically [13]. Finally, this visual property strongly impacts our aesthetic judgments [14, 15] and is often linked to beauty, for instance in the case of faces [16].

Adults detect three main types of symmetry: mirror, translational, and rotational symmetry. In mirror symmetry, half of the design is projected onto the other half, as if reflected on a mirror. In translational symmetry, the design is transposed—repeated without mirroring—one or more times along an axis, while in rotational symmetry a pattern is rotated on its own axis. However, these symmetry types are not equally salient to the human visual system (see [7] for a review): humans appear to be especially sensitive to mirror symmetry, particularly along the vertical axis [17, 18].

The human preference for symmetry is also apparent in our creative productions. When Szilagyi & Baird [19] asked participants to arrange square items into “visually pleasing” one-, two-, or three-dimensional displays, they observed that participants created mostly symmetrical designs. In a similar vein, Westphal-Fitch and colleagues [20] presented participants with images of mosaic-like tiles ordered randomly and asked them to rearrange the array to their liking, without further instruction. The majority of the resulting patterns were highly ordered, and over 70% of them symmetrical, including mirror, rotational and translational symmetries. Adults thus perceive and produce symmetry spontaneously, even when not prompted to do so.

Not only is symmetry a salient visual property, but it also helps the visual system to recognise objects [21, 22], segregate figures from the background [23], and it impacts visual search efficiency [13]. Thus, after a single short view of a novel three-dimensional object, participants recognise the new object—presented rotated—significantly better if it is bilaterally symmetrical rather than asymmetrical [22]. Similarly, participants detect symmetrical two-dimensional shapes embedded in a noisy background (i.e. arrays of Gabor elements) significantly better than asymmetrical shapes [23]. Symmetry relations amongst the elements in a scene are processed in parallel and can facilitate or slow visual search efficiency: it is harder to detect a vertically symmetric target when it is presented with distractors that are also symmetrical along the vertical axis, as compared with distractors symmetrical along an oblique axis [13].

The developmental origins of humans’ perception of symmetry are not well understood. To date, only a handful of studies have explored human symmetry perception in early infancy, using simple shapes, arrays of dots, or patterns, which were always monochromatic. The perception of symmetry in more complex multi-featured stimuli has not been investigated so far for young infants.

The few available studies report that at 4 months of age—the earliest age tested to date—infants discriminate between asymmetrical shapes and shapes with vertical mirror symmetry, i.e. in which the left half of the design is mirrored onto the right half [24]. They also look longer to arrays of dots arranged in vertical mirror symmetry presented side-by-side with asymmetrical or horizontally symmetrical arrays [25], and habituate faster to a simple visual pattern symmetrical along the vertical axis as compared with similar patterns arranged along the horizontal or oblique axes, or asymmetrically [26, 27]. Although 4-month-old infants discriminate between shapes with vertical and horizontal symmetries, they seem to fail to distinguish horizontally symmetrical and asymmetrical shapes [24]. These studies thus suggest a processing advantage for vertical bilateral symmetry, similar to that attested in adulthood [17, 18]. The salience of vertical symmetry might be particularly acute in mirror symmetry, since infants habituate faster to vertical mirror symmetry than to vertical translational symmetry [27]. It remains to be tested whether infants discriminate between asymmetrical designs and designs with vertical translational symmetry. These pioneering studies indicate that an ability to detect symmetry in simple, monochromatic designs appears to be present in early infancy.

Importantly, infants do not navigate a visual world composed of simple one-dimensional stimuli. Instead, they face a complex environment in which stimuli contain multiple features (e.g. colour, shape, etc.) that co-occur in space. And yet, despite the well-established role of symmetry in adult visual perception, it remains unexamined whether infants can detect symmetry in multi-featured stimuli. Moreover, all previous studies presented infants with shapes and arrays that had perfect surface symmetry. Whether infants are able to discriminate symmetrical *structures* lacking perfect surface symmetry from asymmetrical ones is however unknown. While providing infants with perfectly mirrored symmetrical images allows infants to discriminate symmetrical and asymmetrical images by relying on low-level visual mechanisms, presenting them with structurally symmetrical but superficially imperfect sequences might lead them to parsing their structure instead. Thus, in the present study we investigated—for the first time—whether young infants at 7 months of age detect symmetrical structures in elaborate visual sequences, specifically, in multi-coloured mosaic-like abstract visual patterns, to determine whether infants’ ability to detect symmetry is maintained or disrupted in the absence of perfect surface symmetry. This work is therefore exploratory in nature.

**Fig 1:**
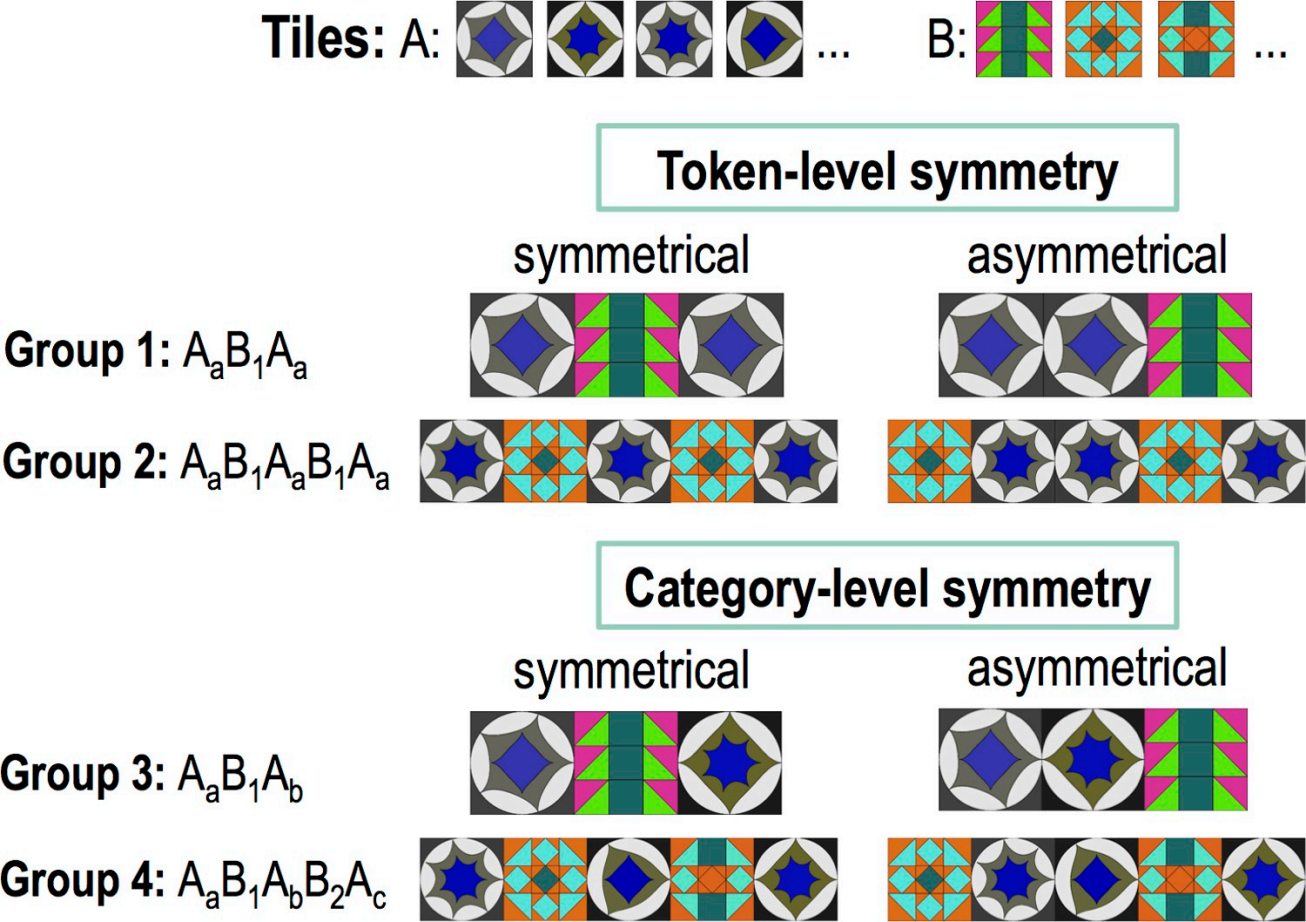
Stimuli. The upper panel depicts a sample of the 18 tiles used to create the mosaics, 9 per category (A or B). The middle panel depicts a sample of the mosaic-like sequences with token-level structural symmetries, and the lower panel of the mosaics with category-level structural symmetries.

The mosaics consisted of colourful square tiles from two distinct types or categories, based on both the shape of their internal elements and their colour combination (Fig 1). These tiles were arranged into horizontal sequences, either in asymmetrical order or in vertical symmetry. We presented 7-month-old infants with multiple instances of structurally symmetrical and asymmetrical mosaics and measured their spontaneous looking times at the two types of patterns. We chose to examine 7-month-olds, as at this age infants are sensitive to contrasts in shape and colour and, importantly, it is the youngest age at which infants are known to group stimuli into larger perceptual units on the basis of form similarity [28]. Interestingly, adults’ perception of symmetry is facilitated in patterns containing elements grouped into clusters [10, 29].

To determine the robustness of symmetry detection in early infancy, we manipulated two aspects of our stimuli, namely the length of the mosaic-like sequences and their level of abstractness, determined by the elements over which the structural symmetry holds (Fig 1). The mosaics were built from the concatenation of either 3 or 5 tiles, and were structurally symmetrical at either the token- or the category-level, i.e. symmetrical at the level of the specific tiles used (repeating a tile), or at the more abstract level of tile category, with no repetition of specific tiles. Symmetrical mosaics had an underlying structure ordered in vertical mirror symmetry: 5-tile sequences had an ABABA structure, 3-tile sequences an ABA structure, where A and B represent the two categories of tiles. In both 3- and 5-tile sequences, the central A element aligns with the vertical axis of the sequence, and the left and right halves of the structure are mirror projections. In summary, stimuli with token-level symmetries repeated specific tiles, while category-level symmetries involved only tile-type, with no repetition (see Fig 1). Importantly, none of the mosaics had perfect surface mirror symmetry. All prior studies examined infants’ perception of surface symmetry. The present study is hence the first one to examine whether infants detect structural symmetry in the absence of perfect surface symmetry.

We predict that differences in looking times to the structurally symmetrical and asymmetrical patterns will reveal infants’ visual preferences. Predicting the direction of infants’ responses is, however, not straightforward. Infants might look longer to the symmetrical patterns if they prefer structural symmetry as a visual property in complex visual sequences. Indeed, previous studies have shown that infants, children, and adults look longer at symmetrical than asymmetrical patterns when these are presented side-by-side (infants at 4 months: [25], but see also [26]; infants at 12 months: [26]; 3- to 6-year-old children and adults: [30]. However, when presented with one display at a time, 4-month-old infants habituated faster to symmetrical patterns and hence looked longer during the presentation of asymmetrical designs [26]. Since our stimuli were similarly presented one display at a time, we predicted longer looking times to structurally asymmetrical mosaics.

Finding that infants detect structural symmetry in our complex stimuli, which are the most ecological stimuli presented in such experiments to date to young infants, will further our knowledge of their pattern parsing abilities. It will also overcome two important limitations of previous studies. First, these early works had very reduced simple sizes. Furthermore, their conclusions were drawn on the basis of pairwise comparisons (it is not reported whether they were corrected for multiple comparisons), rather than on Analyses of Variance across groups or measurements, with one exception [25]. Our study will provide a methodologically and statistically more solid basis for exploring young infants’ perception of symmetry in complex patterns.

## Methodology

### Participants

Ninety-eight 7-month-old infants participated in the experiment (51 girls; mean age: 7;02; SD: 13 days; age range: 6;14–8;04). All infants were born full-term and were being raised around the Paris area in France. Participants were randomly sorted into four groups that differed only in the stimuli they saw during the study, as described in the next section. Thus, 25 of the infants were included in Group 1 (14 girls; mean age: 7;02; SD: 14 days; age range: 6;15–8;04), another 24 infants participated in Group 2 (11 girls; mean age: 7;01; SD: 12 days; age range: 6;15–8;00), 24 infants took part in Group 3 (16 girls; mean age: 6;29; SD: 13 days; age range: 6;14–8;02), and the remaining 25 infants took part in Group 4 (10 girls; mean age: 7;05; SD: 13 days; age range: 6;15–7;27). Data from 17 additional infants were not included due to fussiness or crying (6 infants in Group 1, 8 in Group 2, 1 in Group 3, and 2 in Group 4), and 1 due to equipment failure (Group 4). All parents gave informed consent before their infant’s participation.

The previous studies examining young infants’ surface symmetry detection abilities do not report effect sizes or other statistics that would allow us to run a power analysis. Sample size was hence decided on the basis of infant availability and a recent study by Oakes [31], examining the trade-off between sample size and statistical power in infant looking-time research. This study showed that sample sizes below 24 infants can be underpowered. We thus aimed to include at least 24 infants in each group.

#### Stimuli

Stimuli were 18 square-shaped, multi-coloured tiles of identical size, akin to those used in [32]. Tiles were split into two categories: tiles in category A contained a rounded shape and were coloured black, brown and blue, while tiles in category B contained angular shapes and were coloured red/orange/pink and green (see Fig 1). We combined the A and B tiles into mosaic-like sequences of two types: structurally symmetrical and asymmetrical. The symmetrical sequences followed a simple rule of strict alternation and had two possible lengths: 3 tiles (i.e. ABA) or 5 tiles (i.e. ABABA). All resulting sequences had an underlying bilaterally symmetric structure along the vertical axis. Sequences could be structurally symmetric either at (1) the token level, i.e. each sequence contained a single A and B token: A_a_B_1_A_a_ (Group 1) or A_a_B_1_A_a_B_1_A_a_ (Group 2), or (2) at the category level, i.e. sequences contained different tokens of the same category: A_a_B_1_A_b_ (Group 3) or A_a_B_1_A_b_B_2_A_c_ (Group 4; see Fig 1). While conceptually these stimuli represent bilateral mirror symmetry along a central vertical axis, visually their surface symmetry was not perfect. In the case of mosaics with category-level symmetry this is a necessary result of their creation (because tiles in corresponding positions are different). In mosaics with token-level structural symmetry, the stimuli are more nearly symmetrical, but still violate visual mirror symmetry at a fine-grained level of detail, by copying rather than mirroring the repeated A and B tiles (see Fig 1). Prior studies show that infants detect surface mirror symmetry [24–27]. The present study examines whether infants also detect structural mirror symmetry in the absence of perfect surface symmetry. If infants differentiate between structurally symmetric and asymmetric sequences, this would indicate that they are processing the underlying conceptual symmetry of the symmetric patterns. Alternatively, they might be applying a global level of parsing that disregards these small-scale deviations from perfect visual symmetry.

In order to create asymmetric variants of these mosaics, we switched the order of a pair of adjacent tiles within the sequences, ensuring that all possible orders occurred with the same frequency. The 8 ABA sequences were reordered into 4 BAA and 4 AAB asymmetric sequences (the two underlined tiles are swapped). The 8 ABABA sequences resulted in 2 BAABA, 2 AABBA, 2 ABBAA and 2 ABAAB sequences. The exhaustive combination of the two manipulated parameters, sequence length (3 or 5) and level of symmetry (category or token), generated 4 final sets of mosaics, each containing a total of 8 symmetrical and 8 asymmetrical sequences. Each of these 4 sets was tested in a different group of infants (Groups 1–4, see details below).

#### Procedure

The study took place at the Babylab of the Integrative Neuroscience and Cognition Center (CNRS & Université Paris Cité) in Paris, France, and was approved by the CERES ethics board (Université Paris Cité). Infants were seated on a parent’s lap in a sound-attenuated room with dim lights. A video camera placed above the screen recorded the session. Caregivers wore opaque glasses, preventing them from viewing the stimuli, in order to avoid potential parental influence on the infants. An experimenter—placed outside the testing booth and blind to the stimuli—monitored infants’ looking behaviour and controlled stimulus presentation. Stimuli were displayed using Habit X.10 software [33], on a 23” LCD monitor. Horizontally, the mosaics covered the total width of the screen. Consequently, 3-tile mosaics occupied greater vertical space than 5-tile mosaics on screen, as both types of sequences were generated using the same square tiles.

Test consisted of 16 trials: 8 contained structurally symmetrical sequences and the remaining 8 contained structurally asymmetrical sequences. Although this number of trials is rather high for studies with such young infant populations, we reasoned that manipulating the mosaics’ length and level of structural symmetry could result in differing trajectories of symmetry detection. We nonetheless designed the first 8 trials to contain 4 symmetrical and 4 asymmetrical sequences to allow for the assessment of looking preferences over fewer trials in this initial period. Order of presentation was additionally pseudorandomized so that no more than two trials of the same type occurred consecutively. Trial order also varied across babies.

Infants in Group 1 saw 3-tiled sequences with token-level structural symmetry. Infants in Group 2 saw 5-tiled sequences with token-level structural symmetry. Group 3 was presented with 3-tiled sequences with category-level structural symmetry. Finally, Group 4 saw 5-tiled sequences with category-level structural symmetries (see Fig 1).

**Fig 2:**
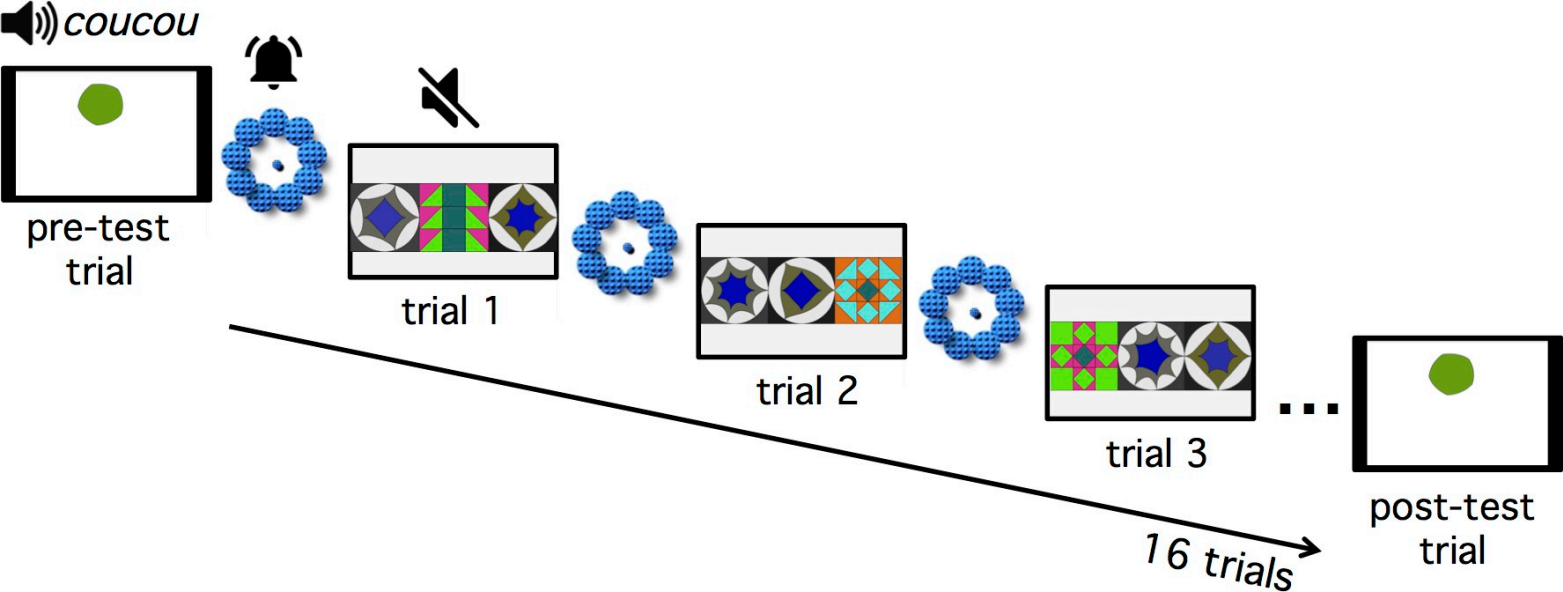
Procedure of the test. The study started with a pre-test trial in order to engage infants’ attention. Infants then saw a total of 8 structurally symmetrical and 8 structurally asymmetrical sequences, which were intermixed and preceded by an attention getter. A post-test trial identical to the pre-test trial ended the study.

The study started with a pre-test trial—a looming ball that changed colour accompanied by a woman’s voice saying “*coucou*”—in order to attract the infant’s attention. Furthermore, each trial began with another attention-getter, i.e. a video showing flashing balls accompanied by a bell sound (see Fig 2). Once the infant looked at the screen, this video disappeared and was replaced by one of the mosaics, which was presented in silence. Infants saw complete stimuli—i.e. all tiles within a given sequence were presented simultaneously—which remained on screen for maximally 30 seconds or until the infant looked away for more than 2 seconds. After this, a new trial began. A post-test trial—identical to the pre-test trial—followed test.

#### Data analysis

To test whether infants discriminated the structurally symmetrical and asymmetrical mosaics, we recorded their spontaneous attention to the screen during the 16 test trials and coded their looking behaviour off-line. Two research assistants, blind to the conditions, coded half of the infants each. In addition, both assistants coded 8 randomly chosen infants, to measure the reliability of their coding. Coders achieved a high level of agreement (r = .96; p < .001). As is customary in studies analysing infant looking behaviour, we excluded from analysis all trials with very short (<1sec) looking times [34]. After applying this criterion, only infants that had a minimum of three trials per condition—structurally symmetrical and asymmetrical mosaics—were retained for analysis. Implementation of these criteria did not result in the exclusion of any babies from analysis. Of the total of 1568 trials (98 infants x 16 trials each), 1530 entered analysis. The remaining 38 trials (2.42%) were excluded due to: (1) having looking times shorter than 1sec (30 trials), (2) experimenter error during online coding (2 trials), (3) and parental interference (6 trials). The sample of 98 infants had a mean number of 7.78 symmetrical trials out of 8 (range 5 to 8) and 7.84 in asymmetrical trials (range 5 to 8). The full set of data is available in the S1 File.

We analysed infants’ average looking times to the structurally symmetrical and asymmetrical trials and statistically evaluated the effects of structural symmetry, sequence length, level of symmetry and variability in looking times. In order to detect potentially different trajectories across groups, we split the test phase into two halves, as is often done in the literature (e.g. [35–38]).

## Results

**Fig 3:**
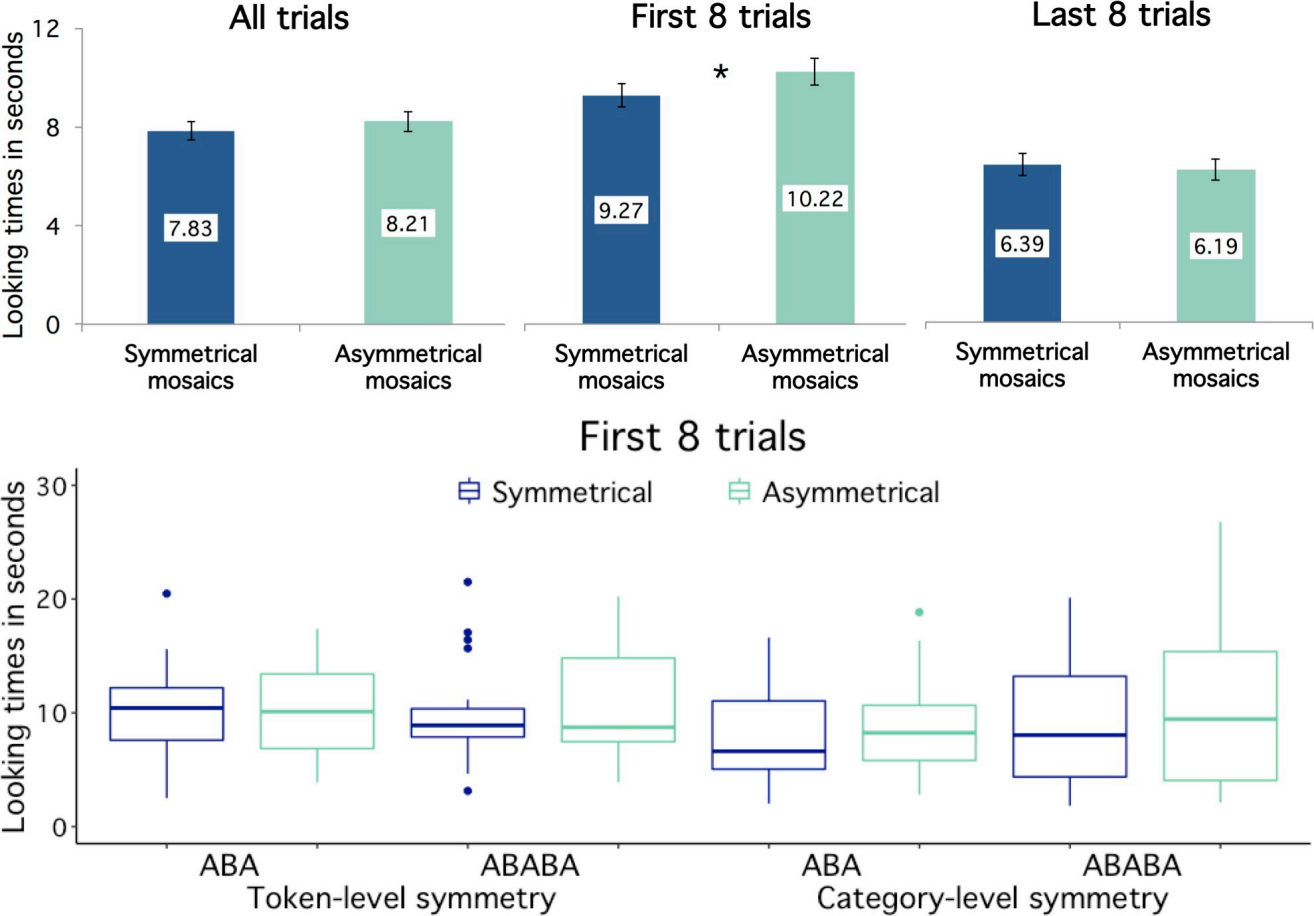
Looking time results. The upper bar plot shows infants’ mean looking times to the mosaics: during the 16 trials (left), in the first 8 trials (centre), and in the last 8 trials (right). Looking times in all 4 conditions (i.e. to 3- and 5-tiled mosaics, with token- and category-level structural symmetry) are collapsed. The y-axis displays the infants’ looking times in seconds. Dark blue bars depict mean looking times to structurally symmetrical mosaics, while light aquamarine bars display mean looking times to structurally asymmetrical mosaics. Error bars represent the standard error of the mean, and statistically significant comparisons are marked with an asterisk. The lower box-and-whisker plot depicts the distribution of infants’ mean looking times per condition during the first 8 trials.

We averaged the infants’ looking times across all trials of the same condition—i.e. structurally symmetric or asymmetric—during the first and second halves of the study (see Fig 3 and Table 1), and carried out a repeated-measures ANOVA with looking time as the dependent variable, Stimulus Type (structurally symmetric or asymmetric) and Block (first 8 trials vs. last 8 trials) as within-subject variables, as well as Sequence Length (3 or 5 tiles) and Symmetry Type (token- or category-level) as between-subject variables. The ANOVA yielded a significant main effect of Block (F(1, 94) = 55.101, p < .001, ŋ_p_^2^ = .370, 95% CI of the difference [2.53, 4.38]), due to longer overall looking times during the first than in the second half of the study. In addition, the ANOVA revealed a significant interaction between Block and Stimulus Type (F(1,94) = 4.801, p = .031, ŋ_p_^2^ = .049). No other effects or interactions reached significance (all *p*s ≥ .085, the results of the ANOVA are detailed in the S2 File).

**Table 1:** Looking time results.

**ALL 16 TRIALS**					
		**Symmetrical mosaics**		**Asymmetrical mosaics**	
		**mean**	**SE**	**mean**	**SE**
**token-level symmetry**	Group 1: ABA	8.90	0.73	8.64	0.81
	Group 2: ABABA	7.83	0.75	8.68	0.83
**category-level symmetry**	Group 3: ABA	7.09	0.75	7.17	0.83
	Group 4: ABABA	7.50	0.73	8.34	0.81
mean of all groups		7.83	0.37	8.21	0.41
**FIRST 8 TRIALS**					
		**Symmetrical mosaics**		**Asymmetrical mosaics**	
		**Mean**	**SE**	**mean**	**SE**
**token-level symmetry**	Group 1: ABA	10.15	0.90	10.49	1.09
	Group 2: ABABA	9.83	0.92	10.63	1.11
**category-level symmetry**	Group 3: ABA	8.15	0.92	8.92	1.11
	Group 4: ABABA	8.94	0.90	10.85	1.09
mean of all groups		9.27	0.46	10.22	0.55
**LAST 8 TRIALS**					
		**Symmetrical mosaics**		**Asymmetrical mosaics**	
		**Mean**	**SE**	**mean**	**SE**
**token-level symmetry**	Group 1: ABA	7.64	0.87	6.79	0.87
	Group 2: ABABA	5.82	0.89	6.73	0.88
**category-level symmetry**	Group 3: ABA	6.02	0.89	5.42	0.88
	Group 4: ABABA	6.07	0.87	5.83	0.87
mean of all groups		6.39	0.44	6.19	0.44

Mean looking times and standard error of the mean (SE) in seconds, to structurally symmetrical vs. asymmetrical mosaics, in the four groups of infants. The upper panel displays mean looking times including all 16 trials, the central panel contains mean looking times to the first 8 trials only, and the lower panel displays mean looking times during the last 8 trials.

In order to explore the significant interaction between block and stimulus type, we carried out separate ANOVAs on the first and last 8 trials of the study (see Fig 3 and Table 1), with Stimulus Type, Sequence Length and Symmetry Type as variables. The ANOVA on the first 8 trials of the study revealed a main effect of Stimulus Type (F(1, 94) = 5.498, p = .021, ŋ_p_^2^ = .055, 95% CI of the difference [0.15, 1.76]), due to longer overall looking times to structurally asymmetrical than symmetrical mosaics. There were no further effects or interactions (all *p*s ≥ .255). In turn, the ANOVA on the last 8 trials of the study revealed no significant effects or interactions (all *p*s ≥ .121). The results of the two ANOVAs are reported in the S2 File.

Our results indicate that 7-month-old infants discriminated structurally symmetrical from asymmetrical mosaics, although this effect disappeared as infants’ attention—as measured by looking time—declined during the course of the experiment. Infants thus appear to have perceived stimulus structural symmetry, and their looking behaviour was not modulated by two additional dimensions of variability present in the mosaics, i.e. stimulus length and symmetry type. Note that, across groups, mosaics also differed in a third source of variability as a necessary consequence of the first two, i.e. the number of unique tiles present in each mosaic. Mosaics with token-level structural symmetries—A_a_B_1_A_a_ and A_a_B_1_A_a_B_1_A_a_—consisted of two unique tiles, one per category (i.e. A_a_ and B_1_). Meanwhile, mosaics with category-level structural symmetries—A_a_B_1_A_b_ and A_a_B_1_A_b_B_2_A_c_—contained either 3 (i.e. A_a_, A_b_, B_1_) or 5 unique tiles (i.e. A_a_, A_b_, A_c_, B_1_, B_2_). In order to determine whether this specific source of variability impacted infants’ discrimination of the structurally symmetrical and asymmetrical mosaics, we ran an additional repeated-measures ANOVA with Number of Unique Tiles (2, 3 or 5) as a between-subjects factor, Stimulus Type (symmetric or asymmetric) and Block (first 8 trials vs. last 8 trials) as within-subjects factors, and looking times as the dependent variable. Once again, a main effect of Block obtained (F(1, 95) = 49.114, p < .001, ŋ_p_^2^ = .341, 95% CI of the difference [2.46, 4.40]), as well as an interaction between Block and Stimulus Type (F(1, 95) = 6.024, p = .016, ŋ_p_^2^ = .060), but no effect of Number of Tiles (p = .306) or interaction (all *p*s ≥ .159, the results of the ANOVA are reported in the S2 File).

## Discussion

We investigated whether young infants perceive structural symmetry in multi-featured abstract visual patterns, presenting 7-month-old infants with images of colourful mosaics built from square tiles from two categories—A and B—based on their colour and internal shape. Tiles were arranged into mosaic-like sequences with a structurally symmetrical (e.g. ABA, ABABA) or asymmetrical structure (e.g. AAB, ABAAB). We measured infants’ spontaneous looking behaviour to both types of mosaics, manipulating two properties of the tile-sequences, namely their length and abstractness of symmetry. Mosaics could consist of sequences of either 3 or 5 tiles (e.g. ABA or ABABA), and be structurally symmetrical at either the token level (i.e. the individual tokens were identical) or at the category level (i.e. only the types of tokens were the same). While the symmetrical sequences had mirror symmetry at the structural level, neither token nor category level mosaics had perfect surface symmetry.

Previous literature examining infants’ perception of symmetry used simpler designs than our multi-featured mosaics, such as arrangements of a few dots, or a simple shape or pattern, all of them mono-chromatic [24–27] and with perfect surface symmetry, making our study with its complex and colourful stimuli exploratory in nature, and the first one to examine whether infants detect structural symmetry in the absence of perfect surface symmetry. We opted to present infants with 16 trials, reasoning that manipulating the length of the mosaics and/or their level of structural symmetry could result in differing trajectories of symmetry detection. Analysis of the 16 trials uncovered no significant difference in infants’ looking times to the structurally symmetrical and asymmetrical mosaics. However, infants’ attention decayed significantly as the study progressed, which suggests that the length of the study was excessive for such young infants. In addition, we observed a significant interaction between infants’ looking times to the structurally symmetrical and asymmetrical sequences and block (1^st^ vs. 2^nd^ half of the study). Therefore, we analysed infants’ looking behaviour during the first and last 8 trials—i.e. the first and second half—of the study separately, and found that during the first half of the study infants looked significantly longer to the structurally asymmetrical mosaics. Neither the length of the mosaics nor their level of structural symmetry modulated infants’ looking behaviour, and we found no significant effect of a third source of variability inevitably present in our stimuli, namely the number of unique tiles present in each mosaic. Our results thus show that 7-month-old infants discriminated between structurally symmetrical and asymmetrical abstract patterns, despite the fact that the mosaics did not have perfect surface symmetry.

The fact that our infants looked longer at the structurally asymmetrical mosaics is consistent with the similar pattern found by Bornstein and colleagues [26], who presented 4-month-old infants with one symmetrical and one asymmetrical image (the symmetrical image having perfect surface mirror symmetry), both monochromatic, each displayed in a different session. Bornstein and colleagues interpreted the shorter looking times to the symmetrical design as evidence that infants process vertically symmetrical patterns more efficiently than asymmetrical designs (or designs with symmetry along other axes), and habituate to them more rapidly. Interestingly, the handful of studies available in the literature suggests that when the images are presented side-by-side, infants look longer to symmetrical patterns instead [25, 26, 30]. The occurrence of both patterns in the literature, seemingly determined by methodological choices, makes it unclear whether the longer looking times to asymmetrical mosaics found in the present study reflect infant’s preference for structurally asymmetrical stimuli, or habituation to symmetrical ones. The direction of the preference notwithstanding, we can conclude that infants detected the difference in structural symmetry, and discriminated between symmetrical and asymmetrical patterns.

The fact that infants’ looking behaviour was not modulated by the length of the mosaics, the level of abstraction of their structural symmetry, or the number of unique tiles per mosaic suggests an automatic and robust detection of structural symmetry in early infancy. The mosaics employed in the present study are, to our knowledge, the most complex abstract stimuli used to test young infants’ perception of symmetry to date. Their complexity lies in the fact that their building blocks vary in both their colour scheme and the shape of their sub-elements, and the composite stimuli thus contain multiple features that infants could potentially process.

Whether the infants accessed the internal structure of the overall stimulus, or whether they instead perceived the structurally symmetrical mosaics as wholes or Gestalts cannot be directly determined from our study. Superficial features such as colour might have sufficed for infants to perceive the whole image as a unified symmetrical object, a Gestalt, without further parsing the mosaics’ internal structure. Alternatively, infants might instead have built an abstract representation of the tiles establishing the sequences’ overall structure. For instance, the colour contrast of the A and B tiles might have allowed infants to discover the two tile categories—e.g. blue-brown-black tiles vs. orange/red/pink-green tiles—and detect the mirror symmetry characterising stimulus structure based on colour. Shapes could have worked similarly. Both accounts predict the pattern of results obtained, in which the level of symmetry—category or token—did not modulate infants’ looking behaviour. Moreover, these two hypotheses are not mutually exclusive, and infants at this age may be able to perceive symmetry in surface features *and* parse the structure of the sequences. Further research will be needed to resolve this issue, but both of the accounts allow us to conclude that infants at this age are sensitive to structural symmetry in complex visual images.

Although more research will be needed to resolve this issue, by 7 months of age, infants are able to parse structure both in the auditory and visual domain, including in abstract visual stimuli (see [39] for a review). In the last two decades, a wealth of research has investigated the learning mechanisms that allow infants to learn rules, that is, to detect abstract patterns or relations between elements in a set of inputs, and generalise these relations to new items. A substantial part of this work has focused on structures containing repetitions, as the identity relation is arguably the simplest abstract rule [40]. Indeed, at 7 months of age—the same age as our participants—infants readily learn structures implemented over syllables containing both adjacent (e.g. ABB: *ba po po*) and non-adjacent repetitions (e.g. ABA: *ba po ba*) [40]. After a short familiarization with one of the structures (e.g. ABB: *ba po po*, *ga ti ti…*) infants can readily discriminate new, previously unheard tokens instantiating the familiar rule (ABB: *wo fe fe*) from tokens instantiating a novel rule (e.g. ABA: *wo fe wo*).

The body of research following up on Marcus and colleagues [40] mainly focused on speech stimuli. However, several studies have examined whether infants’ rule-learning abilities can also be observed in the visual domain [41–45], revealing that rule learning is not exclusive to speech processing. Infants are also able to learn repetition-based rules from abstract visual stimuli but, interestingly, their abilities appear to be strongly modulated by how the stimuli are presented. Although infants at 7- and even 3-months of age successfully discriminated sequences of coloured geometrical shapes containing adjacent (ABB: *grey octagon*, *red square*, *red square*) and non-adjacent repetitions (ABA: *grey octagon*, *red square*, *grey octagon*) when the individual shapes of each triad are displayed sequentially from left to right on the screen [41, 42], 7-month-olds failed when the same sequences are presented from right to left [41]. Furthermore, when the geometrical shapes are displayed one-by-one at the centre of the screen, 5-month-old infants failed to discriminate the ABB and ABA sequences [43], and 8-month-old infants succeed only under specific conditions (when first habituated with sequences containing an adjacent repetition at the right edge: ABB vs. ABA, but fail if habituated with initial adjacent repetitions: AAB vs. ABA, or with non-adjacent repetitions: ABA vs. ABB; [44]).

The abstract visual stimuli used in this previous work have a structure similar to that of our 3-tiled mosaics (i.e. ABB, ABA), but crucially, the shapes within sequences were presented sequentially, i.e. one at a time, as is typical in the rule-learning literature (with the sole exception of [42]). Interestingly, Endress and colleagues propose that adjacent repetitions in speech are a perceptual primitive detected automatically by the perceptual system [46]. These authors argue that infants might discriminate the ABB and ABA structures by automatically detecting adjacent repetitions, without having built an abstract representation of the ABB and ABA structures. It remains to be determined whether adjacent repetitions are similarly perceptual primitives in visual sequences presented sequentially (as in the rule-learning literature), as well as simultaneously as in our stimuli.

Endress and colleagues [46] propose that sequence edges are also a perceptual primitive, and argue that item position within sequences is encoded relative to their edges. In half of our 5-tiled structurally asymmetrical mosaics (i.e. those with AABBA and ABBAA structures) tiles were repeated at sequence edges (and also internally). In the remaining half, repeated tiles occurred only sequence-internally (i.e. those with BAABA and ABAAB structures). To rule out a potential influence of edge in infants’ looking behavior, we ran a repeated-measures ANOVA with looking times to asymmetrical mosaics as the dependent variable, Position of Repetition (at edge or not) as within-subject variable, and Symmetry Type (token- or category-level) as between-subject variable. We found no effect or interaction (all *p*s ≥ .264; the results of the ANOVA are reported in the S2 File).

In summary, the results of the present study demonstrate that young infants can detect symmetrical structures in elaborate abstract visual patterns without training and in the absence of perfect surface symmetry. This finding furthers our knowledge of the developmental roots of infants’ symmetry perception, opening the way to new investigations to help elucidate the extent to which infants parse the internal structure of the visual input, and to determine the impact of this salient visual property in other aspects of visual perception.

## Supporting information

S1 File (XLSX)

S2 File (XLSX)
